# Study on the Mechanical Properties and Strengthening Mechanism of Interface-Modified Carbon Fiber Mesh Reinforced Cement-Based Composites with SCA&HMC

**DOI:** 10.3390/molecules24213989

**Published:** 2019-11-05

**Authors:** Bo Wu, Xiaohai Xu, Shigang Luo, Dedao Yan, Kai Song, Xiang Zhang, Fang He

**Affiliations:** 1 School of Materials Science and Engineering and Tianjin Key Laboratory of Composite and Functional Materials, Tianjin University, Tianjin 300350, China; wubo@tju.edu.cn (B.W.); yandedao@gmail.com (D.Y.); songk@tju.edu.cn (K.S.); 2 Carbon Technology Group Co., Ltd., Tianjin 300385, China; 13820253779@139.com (X.X.); luoshigang@whut.edu.cn (S.L.)

**Keywords:** silane coupling agent, hydroxymethyl cellulose, interface bond strength, carbon fiber mesh

## Abstract

Carbon fiber mesh reinforced cement-based composites (CMCCs) have received extensive attention in the field of engineering repair and structural reinforcement due to their outstanding properties such as two-way force, rust prevention, high specific strength, and low base surface requirements. However, the development of this material has been slowed down to some extent due to the poor interfacial bonding between the carbon fiber mesh and the cement matrix. In this paper, a novel fabrication strategy was proposed in which the carbon fiber mesh was modified with epoxy resin and silane coupling agent (SCA) to increase its surface chemical activity. Meanwhile, the hydroxymethyl cellulose (HMC) was also filled into the concrete matrix to improve the mechanical strength of the matrix as well as the load transfer behaviors between the mortar and carbon fiber (CF) mesh. The potential to employ SCA and HMC was evaluated for the making of CMCCs via the above methods. The results showed that the longitudinal shear strength of composites with SCA and SCA&HMC increased by 26.6% and 56.1% compared to those of CF with epoxy resin (EP) reinforced composites, respectively. The flexural strength of composite with SCA&HMC increases by 147.6% compared to I-(F) without CF. The novel II-HCM&CF/EP-SCA composites with excellent performance are promised to be applied in practical uses.

## 1. Introduction

With the rapid development of the construction industry around the world, the number of different buildings based on masonry structure has increased dramatically. On this condition, special attention has been paid to the enormous economic loss caused by destructions, due to the economic change, such as earthquakes, tsunamis, and mudslides [1]. Thereby, it is extremely urgent to solve the safety problems by effectively strengthening the building. In order to increase the strength of the existing masonry buildings and extend their service life, various reinforcement methods, such as the clad steel plate reinforcement method, the clad fiber reinforcement method, and the fiber reinforced cementitious matrix composites reinforcement method, have been used to reinforce the masonry structure [2]. Among them, carbon fiber mesh reinforced cement-based composites (CMCCs), thanks to their outstanding advantages of two-way force, rust prevention, high specific strength, and low base surface requirements have attracted the attention of researchers and industry at home and abroad. For example, Krevaikas et al. showed that the bearing capacity and deformation capacity of the masonry column with rectangular section after reinforcement can be significantly improved in the CMCCs [3]. Li et al. showed that locating the carbon fiber mesh in the center of the carbon fiber reinforced concrete slab could result in significant improvement in the plasticity of the cement-based material [4]. Up to now; however, the lack of efficient interlaminar shear strength between the carbon fiber mesh and the mortar interface is still unsolved and has limited the development of the CMCCs.

It is well known that the interface which transfers the stress from the matrix to the reinforcement up loading plays a key role in the mechanical performance of the composite [5,6]. Roughness and chemical activity are commonly reckoned as the two main factors affecting the interfacial bond strength of composites. The low chemical activity or relatively smooth surface of the carbon fiber mesh result in low bonding strength on the interface between carbon fiber mesh and mortar, which is macroscopically manifested by the poor anchoring effect of the reinforcement system [7]. When the CMCCs are under loading, the internal interface of the system is prone to relative slipping, which causes the safety of the reinforcement system to be reduced and fail [8,9,10]. Some advancements have been made in the surface modification to improve the interfacial bonding strength. From one aspect, Thomas et al. improved the surface roughness of carbon fibers by introducing the free radical polymerization of hydroxyethyl methacrylate, which had a significant effect on enhancing the bond strength of the interface [11]. Jacopo et al. showed that interfacial mechanical riveting strength can be achieved by adding different sizes of quartz sand at the interface [12]. From another aspect, the incorporation of polymer coating between fibers and the resin matrix will increase the viscous character of the material through the stick–slip action and the shear deformation occurring on the viscoelastic interlayer–matrix interface [13,14]. For example, Yan et al. proposed that the carbon fibers surface grafting polydopamine can effectively improve the interfacial bond strength of composites [15]. Bowman et al. pointed out that the epoxy sizing agent provided better protection to carbon fibers [16]. With abundant oxygen-containing functional groups, epoxy resin has the advantage of high chemical activity and low cost and it is an ideal choice for the surface modification of carbon fiber. The poor interlaminar strength is an inherent drawback of carbon fiber/epoxy composites [17]. The Si–O functional group of silane coupling agent (SCA) was also verified to react with inorganic substances [18,19]. The hydrophilic group of the silane coupling agent could link with the hydroxy group by an organic covalent bond [20]. Li et al. proved that the interfacial shear strength of composites improved by 43% using the surface coating treatment for aramid fiber by epoxy resin and silane coupling agent [21]. Yuan et al. applied the epoxy resin and SCA, respectively, on the surface of the carbon fibers to improve the micro-interlock between carbon fibers and substrate [22]. Epoxy resin (EP) was modified by the incorporation of SCA to improve the interfacial adhesion between carbon fiber and EP [23,24,25]. However, there is no report on the introduction of SCA into CMCCs for interfacial modification. At present, the research on the bond strength of the interface between carbon fibers and mortar focus on the modification of the carbon fiber surface. However, there have been few reports on the modification of the mortar matrix to increase the bond strength of its interface. Cellulose is of increasing interest due to its appealing inherent properties: The ability to form effective hydrogen bonds across the cellulose chains or within other polymeric matrices [26]. Cheng mentioned that the addition of 8% cellulose increased the ductility and failure stress of the cement system due to its crack-bridging function [27]. Shama et al. showed that carboxymethyl cellulose (CMC) consisted of molecular bonds of water-deficient glucose units with a large amount of hydroxyl groups, which can increase the bond strength between the mortar and carbon fibers, and leads to a significant increase in the mechanical properties of the mortar matrix [28]. However, it is hard to achieve a satisfactory result in improving the mechanical strength of CMCCs using the single modification methods mentioned above. In this work, a combined strategy was proposed in which the chemical activity of a carbon fiber mesh surface was increased mainly by introducing the polar group, and the interface between mortar and carbon fiber fabric was modified by SCA and hydroxymethyl cellulose (HMC). The results demonstrated that chemical bonding was formed to improve the interfacial adhesion effectively, thus positively affecting the mechanical strength of the composites. Besides, the failure modes of the composites were also discussed to reveal the role of interface on the fracture behaviors.

## 2. Research Contents and Methods

### 2.1. Materials

The virgin polyacrylonitrile (PAN)-based carbon fiber mesh was supplied by Carbon Technology Group Co., Ltd., with diameter of fibers of 7 μm and density of 1.8 g/cm^3^. Silane coupling agent (KH560) was purchased from Hubei Blue Sky New Material INC. Hydroxymethyl cellulose (HMC) with viscosity of 100,000 was provided by Source Leaf Biotechnology Co., Ltd. (Shanghai). The epoxy resin with proprietary formulations was obtained from our lab, including bisphenol-A epoxy (128) and polypropylene glycol diglycidyl ether and1,4-butanedioldiglycidyl ether as the matrix material and alicyclic amine and polyetheramine modification as the hardener. Carbon wet spraying mortar (CWSM) was provided by Carbon Technology Group Co., Ltd. All of the double shear tests were carried out on a single type of substrate, namely clay brick. Specifications of materials are presented in Table 1.

### 2.2. Preparation of Specimens

#### 2.2.1. Surface Modification of Carbon Fabric

**Figure 1:**
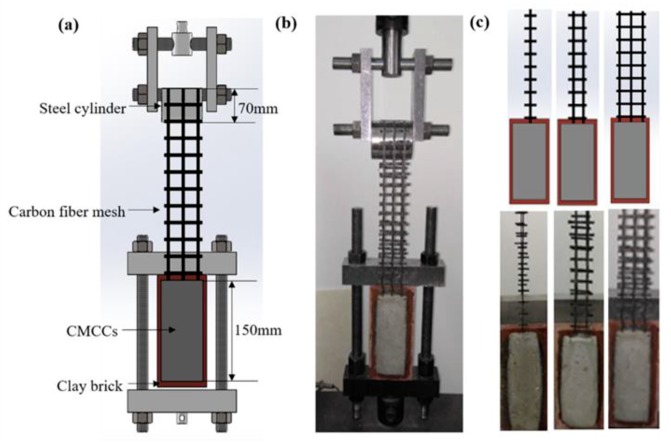
(**a**) Illustration and (**b**) digital image of the double shear test set-up; (**c**) double shear test specimen of CMCCs with different numbers of carbon fiber meshes.

Three different types of carbon fiber mesh with diverse designed stacking size (single, double, triple) were prepared to make CMCCs for double shear test specimens (Figure 1), whereby S, D, T represented the number of carbon fiber meshes with the length of 98 cm. The process of sizing treatment was performed by hand. The virgin carbon fiber mesh was infiltrated with the obtained epoxy resin by a process of extrusion. Then the sized fibers were dried instantly in a hot gas oven at 80 °C/2 h. Meanwhile, the silane coupling agent (SCA) was also applied with the purpose of increasing the chemical activity of the carbon fibers’ surface. When SCA (1%) was added into epoxy resin, the carbon fabric was treated following the same procedure. The above meshes with a size of 40 × 160 mm were cut as reinforcements for the test specimens of flexural properties.

#### 2.2.2. Preparation of the Double Shear Test Specimens

As the cement-based matrix, two different kinds of polymer mortar (I/II) were considered. I represents neat wet spray polymer mortar, II stands for wet spray polymer mortar with HMC. In order to enhance surface roughness, the experimental concrete bricks were polished and cleaned. The epoxy-based interface agent was applied to wet the surface of the concrete bricks. A thin layer (5 mm) of mixed polymer mortar was firstly smeared on the surface of concrete followed by a layer of fabric with a bond length of 150 mm. Finally, the top layer of polymer mortar was prepared with the thickness of 10 mm. The as-obtained samples were cured for 28 days in laboratory conditions of 20 °C and 70% relative humidity. With regard to mortar I, the samples using the carbon fabric before and after being modified by coating of epoxy resin and epoxy resin with SCA were denoted as I-CF, I-CF/EP, and I-CF/EP-SCA, respectively, where I-CF was the control sample. Similarly, the specimens using mortar II and the carbon fabric modified by epoxy resin with SCA was denoted as II-HMC&CF/EP-SCA.

#### 2.2.3. Preparation of the Three-Point Bending Test Specimens

In order to validate the change of the shear strength between the carbon fiber mesh and the mortar layer at multiple angles, the flexural performance tests were conducted. The samples without carbon fiber mesh were fabricated, which was defined as the I-(F). The evenly-mixed mortar I was infiltrated into a mold of 40 × 40 × 160 mm. After, the mold was placed on a vibrating table for discharging air bubbles inside the mortar. Completed samples of I-(F) were wet-cured for 3 days and the mold was verified to be not demolished. Next the samples were wet-cured for 25 days with only the bottom mold left after removing the side mold. On the basis of the above-mentioned sample preparation process, the other four samples were obtained using carbon fiber mesh as a reinforcing material placed at a position 10 mm from the bottom surface. The composite with mortar I fabricated from carbon fabric before and after being modified by coating of epoxy resin and epoxy resin with SCA were denoted as I-CF(F), I-CF/EP(F), and I-CF/EP-SCA(F), respectively. While, the specimens using mortar II and the carbon fabric modified by epoxy resin with SCA was denoted as II-HMC&CF/EP-SCA(F).

### 2.3. Performance Testing and Characterization

#### 2.3.1. Double Shear Tests

The mechanical properties of CMCCs were analyzed by double shear specimen experiments. In this case the load is transferred to the matrix only through the fabric. In order to guarantee that the applied load direction of fabric is parallel to the brick face, the diameter of steel cylinder was designed to be equal to the thickness of the brick plus twice the thickness of the first layer of mortar [9]. The double shear test was carried out on the universal test machine (UTM5105) with a maximum capacity of 100 kN. The model of the load cell of the dynamometer was DBSL-10t with the maximum capacity of 10,000 kg. The specimen was deformed in the test fixture with a test speed of 2 mm/min. A total of twelve sets of double shear test samples were prepared, five for each set, which were I-CF, I-CF/EP, I-CF/EP-SCA, and II-HCM&CF/EP-SCA. The double shear test specimens and the test fixture are shown in Figure 1.

#### 2.3.2. Three-Point Bending Test

**Figure 2:**
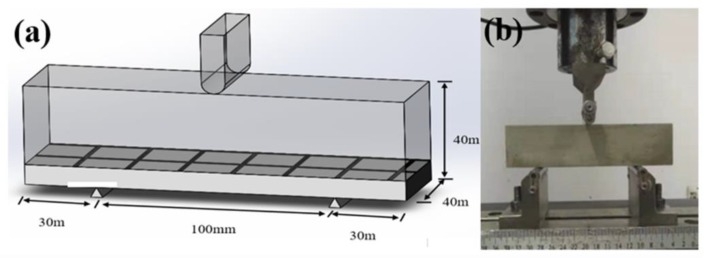
(**a**) Illustration and (**b**) digital image of the flexural performance test set-up.

The flexural property of the composites was measured using the three-point bending test according to GB/T 17671-1999. Flexural performance test specimens and the test fixture are shown in Figure 2. The specimen was placed in the fixture with the test speed of 0.05 kN/min. The flexural performance test equipment was a universal test machine (UTM5105) with a maximum capacity of 100 kN.

#### 2.3.3. Scanning Electron Microscopy Analysis

In order to observe the fracture surfaces of carbon fibers (CFs) and the interface morphology between carbon fibers and polymer mortar, scanning electron microscopy (SEM, equipment type: PHILIPS XL30) was employed at an operating voltage of 10 KV. Due to the sizing agent on the surface of carbon fibers having poor electrical conductivity, specimens were sputtered with an Au coating before testing.

#### 2.3.4. X-Ray Photoelectron Spectroscopy Analysis

The elements and function groups of the carbon fabric presented in the crack were analyzed by X-ray photoelectron spectroscopy (XPS, Axis Ultra DLD) with an Al K alpha source (λ = 0.834 nm, 1486.6 eV).

## 3. Results and Discussion

### 3.1. Double Shear Tests

The experimental data is summarized in Table 2, which includes the type of composites, the number of samples, the load Force (F) measured by the experiment and its average value $F_{av}$, the corresponding coefficient of variation CoV, the average value experimental tensile strength $\sigma_{av}$ during reinforcement, and the tensile ultimate strength (see Table 1) utilization η.

**Figure 3:**
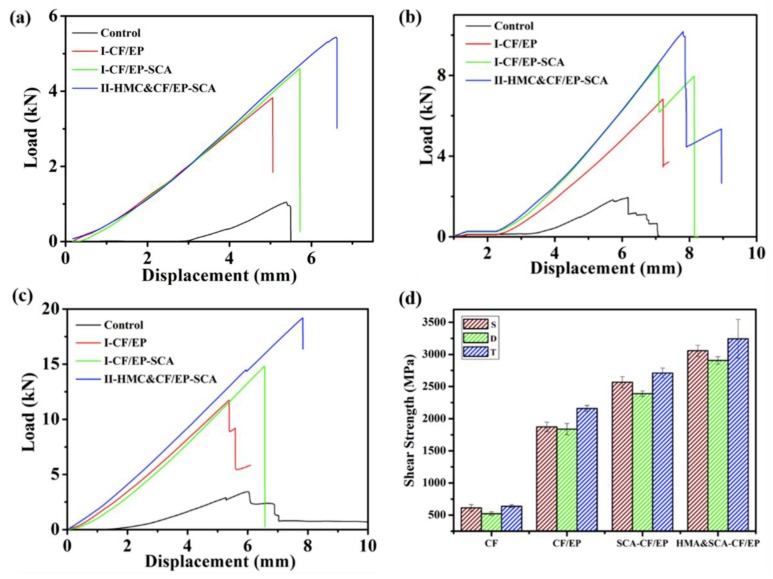
Load–displacement curve of the double shear test (**a**) S, (**b**) D, (**c**) T; (**d**) statistics of the shear strength of CMCCs.

Shear strength refers to the maximum shear stress per unit area when the composite is subjected to shear stress. When shear stress is loaded on composites, the cracks firstly appear at the interface of composites [30], and the longitudinal shear strength could be used as an indicator to compare the interface bond of composites [31,32]. As shown in Figure 3a, the longitudinal shear load average value of I-CF is 1089.2 N, while that of the composites treated by EP increases to 3528.8 N due to the improvement of bonding strength between carbon fiber and mortar matrix. For I-CF, when the load on the composite material reaches the maximum value, it turned to decrease slowly afterwards. This indicates that the carbon fiber mesh slip failure occurred in the test specimen at the time. Compared to the composites I-CF, the load immediately dropped to zero after reaching the maximum shear strength for the I-CF/EP composite. The longitudinal shear load average value of I-CF/EP-SCA is 4563.2 N, which increased by 29.3% compared with that of I-CF/EP. It suggests that SCA has a significant effect on the increase in bonding strength between the carbon fiber and epoxy resin coating. With the HMC added into mortar matrix, the longitudinal shear load average value of II-HMC&CF/EP-SCA increases to 5435.6 N, which is 19.2% higher than that of I-CF/EP-SCA. The result demonstrates that the combination strategy is beneficial for the interface load to transfer uniformly between the carbon fibers and mortar matrix.

For the composites made by the double carbon fiber meshes in Figure 3b and the triple carbon fiber meshes in Figure 3c, the load–displacement curves shared similar trends with the composites made by the single-carbon fiber mesh. For the composites made by double carbon fiber meshes, the curves of I-CF/EP, I-CF/EP-SCA, and II-HMC&CF/EP-SCA had as sawtooth condition as the load value continued to increase. This is because the double carbon fiber meshes are connected by wefts. There will be load transfer inside the matrix when the meshes are under load. It is difficult to ensure a uniform distribution of the system internal load. The load-bearing area will partially fail when stress concentration occurs inside the system. Therefore, as the displacement increases, the curve will have a sawtooth condition until the load-bearing area fails. For the composites made by triple carbon fiber meshes, the curves of I-CF/EP-SCA and II-HMC&CF/EP-SCA without a sawtooth condition indicated that the internal load transfer of the system is uniform. Triple carbon fiber meshes are combined with force and the addition of SCA and HMC increase the bond strength between the carbon fiber mesh and the mortar interface, which is beneficial to the uniform transfer of load. As shown in Figure 3d, the longitudinal shear strength of the composites I-CF/EP using single, double, and triple carbon fiber meshes are 2095 MPa, 1943 MPa, and 2209 MPa, respectively, while the average tensile strength of the composites I-CF/EP is 229.1% higher than that of I-CF. The longitudinal shear strength of the composites (I-CF/EP-SCA) using single, double, and triple carbon fiber meshes are 2565 MPa, 2391 MPa, and 2711 MPa, respectively. The longitudinal shear strength of the composites (II-HMC&CF/EP-SCA) using single, double, and triple carbon fiber meshes are 3077 MPa, 2905 MPa, and 3244 MPa, respectively. Compared with the composites (I-CF/EP), the longitudinal shear strength for I-CF/EP-SCA and II-HMC&CF/EP-SCA increased by 26.6% and 56.1%, respectively.

### 3.2. Scanning Electron Microscope (SEM) Tasks

**Figure 4:**
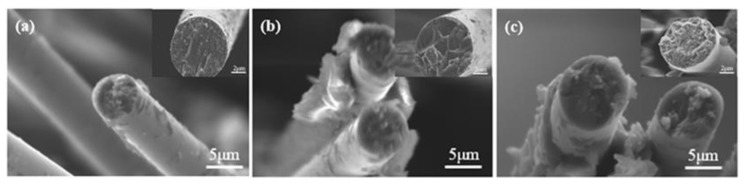
SEM micrographs of the fracture surface of (**a**) I-CF/EP, (**b**) I-CF/EP-SCA, and (**c**) II-HMC&CF/EP-SCA.

SEM characterization was carried out to compare the facture surface and analyze the strengthening effect of SCA and HMC in the composites. As presented in Figure 4a, the CFs surface of I-CF/EP are smooth without coated epoxy. The inset in Figure 4a shows a typical feature of brittle fracture, which indicated a relatively weak interface between CFs and EP. After adding SCA into EP, the residue of EP coated on the surface of CFs could be observed obviously (Figure 4b). On this basis of the sample morphology of II-HMC&CF/EP-SCA in Figure 4c, it was learned that the amount of EP and mineral ion attached to the surface of CFs significantly increase. Compared to I-CF/EP, the fracture morphology of I-CF/EP-SCA and II-HMA&CF/EP-SCA were characterized with fractured dimples from the magnified fracture morphology in Figure 4b,c. A number of bumps existed in the fracture position, suggesting that a high degree of interfacial bonding strength was achieved in I-CF/EP-SCA and II-HMA&CF/EP-SCA.

**Figure 5:**
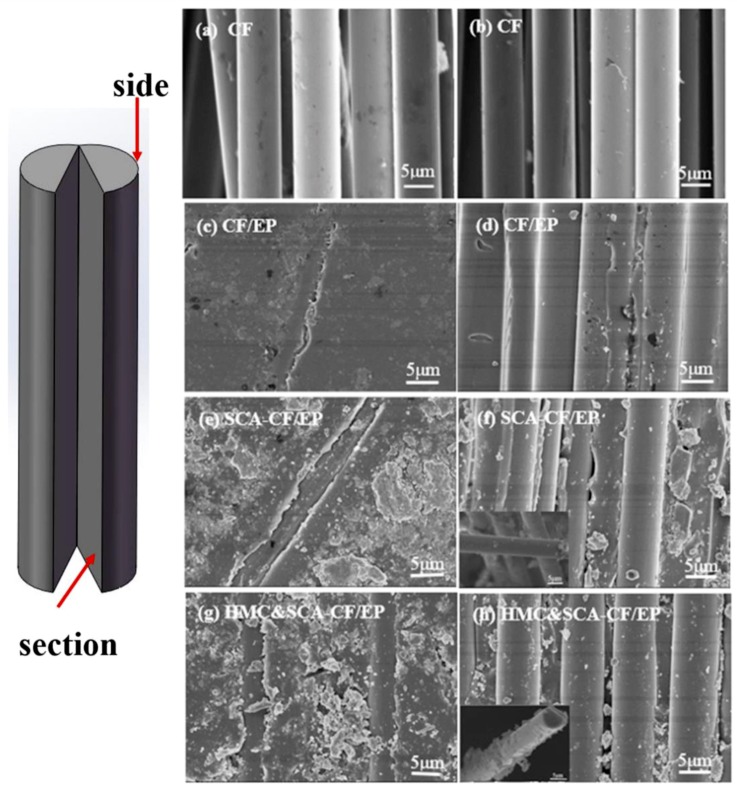
SEM micrographs of the fractured surface of (**a**,**b**) I-CF, (**c**,**d**) I-CF/EP, (**e**,**f**) I-CF/EP-SCA, and (**g**,**h**) II-HMC&CF/EP-SCA.

To further verify the bonding effect between mortar and CFs, the surface morphologies of CFs infiltrated in the mortar matrix were characterized by SEM. For I-CF, Figure 5a shows the smooth surface between CF and mortar, which are conductive to the chemical inertness of the carbon fiber surface. From the perspective of the cross-sectional view of the specimens in Figure 5b, it seems impossible for the CF without epoxy coating to have a good adhesion with mortar. For I-CF/EP, the CF coated by EP can be fixed in mortar with a gap of 1 μm (Figure 5c). Most of the carbon fiber is embedded in the resin, leaving only a small portion outside the matrix, so the carbon fibers have the same dimension in Figure 5c,d. CFs were not uniformly coated with the epoxy coating, which indicates that the bond strength between the epoxy coating and carbon fiber needs to be improved (Figure 5d). For I-CF/EP-SCA, the surface between CF and mortar still has a gap with the addition of SCA in Figure 5e. However, the epoxy resin on the carbon fiber is more evenly distributed. At the same time, the amount of mortar attached to the surface of the carbon fiber is increased in Figure 5f. It is speculated that SCA is helpful in aiding the bond strength between CF and EP. For II-HMC&CF/EP-SCA, the gap between carbon fiber and mortar is significantly narrowed with HMC in Figure 5g. It is advantageous to form an effective chemical connection at the interface of mortar and carbon fiber, since HMC has a large amount of hydroxyl groups. As shown in Figure 5h, much more mortar attached to the surface of the carbon fiber was found compared with I-CF/EP-SCA. The SEM characterization results confirmed that the mechanical properties between CF and mortar were enhanced with different degrees with the addition of SCA and HMC.

### 3.3. X-Ray Photoelectron Spectroscopy Analysis

**Figure 6:**
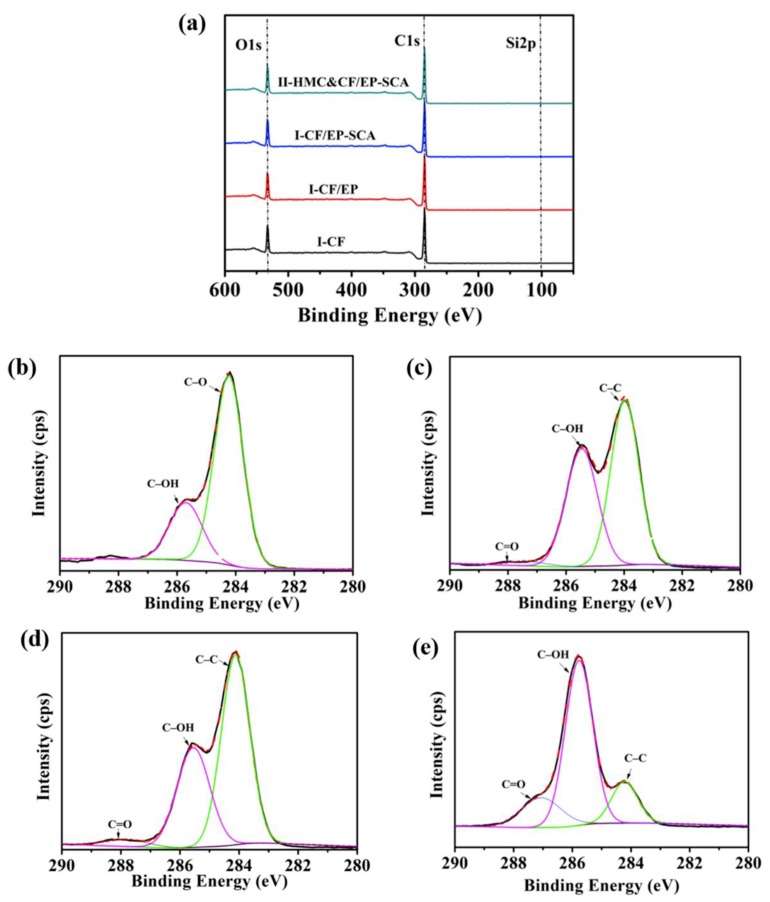
(**a**) XPS spectra and the corresponding C 1s peak-fitting curves of (**b**) I-CF, (**c**) I-CF/EP, (**d**) I-CF/EP-SCA, and (**e**) II-HMC&CF/EP-SCA.

The chemical compositions of the CFs before and after surface modification were measured by X-ray photoelectron spectroscopy (XPS). It can be speculated as to whether the SCA and HMC were successfully coated on the surface of CF by the change of type and content of the element. Figure 6a shows the XPS wide scans of I-CF, I-CF/EP, I-CF/EP-SCA, and II-HMC&CF/EP-SCA. It can be seen that there are significant changes to the surface element content after operating the sizing agent coating. The full XPS spectra of composite showed that main peaks could be indexed to Carbon 1s orbital electron peak (C 1s), O 1s and Si 2p regions (Figure 6a). The content of C on the surface of I-CF/EP decreases slightly but that of O increases moderately, indicating that when the sizing agent is coated, the epoxy group with extremely active chemical activity is successfully introduced on the surface of CFs, compared to I-CF. The SCA contains an epoxy group at the end and a Si–O group inside. Compared with I-CF/EP, I-CF/EP-SCA possesses more element content of Si, which proves that there is a silane coupling agent in the EP coating. For II-HMC&CF/EP-SCA, the element content of O increases again with the falling element content of Si, due to the large amount of hydroxyl of HMC. The changes of the element content on the surface of carbon fibers are shown in Table 3.

To further investigate the chemical compositions of the surface of CFs, C 1s regions of the XPS spectra of I-CF, I-CF/EP, I-CF/EP-SCA, and II-HMC&CF/EP-SCA are evaluated in Figure 6b–e, respectively. For I-CF, the C1s core-level spectrum can be fitted into two peak components: The binding energy of C–C and C–OH were 284.6 and 285.4 eV, respectively. For I-CF/EP and I-CF/EP-SCA, C1s core-level spectrum can be fitted into three peaks, of which a new peak at 287.3 eV associated with C=O was found. The appearance of the C=O peak is mainly caused by the epoxy coating and the silane coupling agent [33,34]. In Figure 6e, the increase of C–OH and C=O peaks is due to the high content of hydroxyl groups in HMC. The bonding intensity increase of C–OH or C–O is related to the dimpled fracture surface of II-HMC&CF/EP-SCA. The increase in the content of C–OH suggested that an increase in the bond strength of the interface between the carbon fiber mesh and the mortar, which further increases the load-carrying capacity of the composites. Therefore, the emergence of the dimpled fracture surface that consumes a lot of energy becomes inevitable.

### 3.4. Failure Analysis

**Figure 7:**
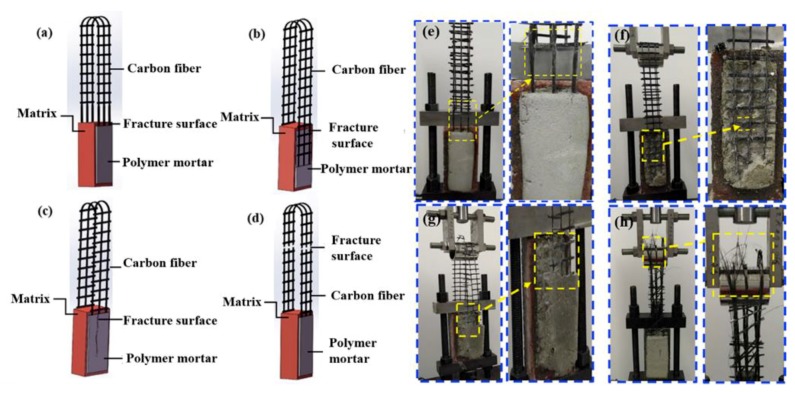
Different failure modes: (**a**,**e**) Fiber slippage; (**b**,**f**) EP–mortar separation; (**c**,**g**) internal damage of mortar layer; (**d**,**h**) fiber rupture.

Bond failure could be observed macroscopically when the specimens were subjected to the ultimate load, which existed in the four types: Carbon fibers sliding inside the matrix, epoxy coating separating from the mortar; cracking of the mortar layer, and carbon fiber fracture outside the mortar covering area. As shown in Figure 7a,e, it is extremely difficult for carbon fibers to form an effective chemical connection and mechanical interlock with the mortar due to the chemically inert nature of its smooth surface. In addition, compared with the size of mortar particles, the size of carbon fiber strands and the distance between strands are too small to achieve effective anchoring effect. Small contact area, low surface roughness, and low chemical activity are the main factors causing fiber slippage. During the mechanical properties test, carbon fiber slippage inside the mortar is a typical failure mode without significant crack propagation, due to its nonimpregnated nature in epoxy. For the carbon fiber bundle with epoxy coating, the probability of slip between the carbon fiber strands is greatly reduced. As shown in Figure 7b,f, epoxy coating separation from the mortar occurred due to the lack of an effective chemical bond between the epoxy coating and the mortar. It is well known that mortar contains a large amount of inorganic substances, making it difficult to be chemically bonded with epoxy resin. The failure from the mortar layer fracturing appears when the chemical connection between the epoxy resin and mortar is rapidly increased, as shown in Figure 7c,g. It proves the feasibility to improve the chemical connection between organic materials and mineral ion, because of the rich reactive groups in SCA. Furthermore, it reveals that the fracture of carbon fibers outside the mortar covering area occurred when the carbon fibers were fixed firmly by the mortar layers, as depicted in Figure 7d,h. CMCCs became an integrated solid structure because the addition of HMC increased the consistency of the mortar and the strength of the mortar systems, indicating the applicability of SCA and HMC for carbon fiber mesh reinforced cement-based composites.

### 3.5. Three-Point Bending Test Results

**Figure 8:**
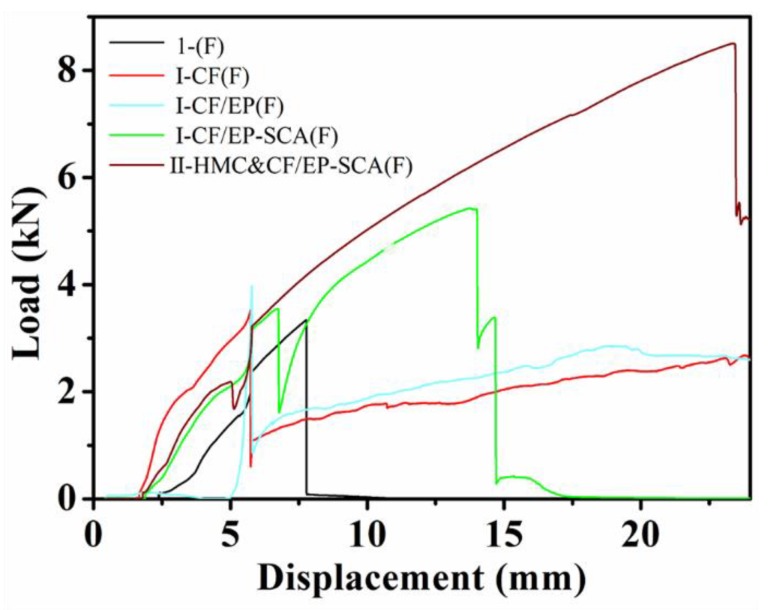
Load–displacement curve of the flexural performance test.

**Figure 9:**
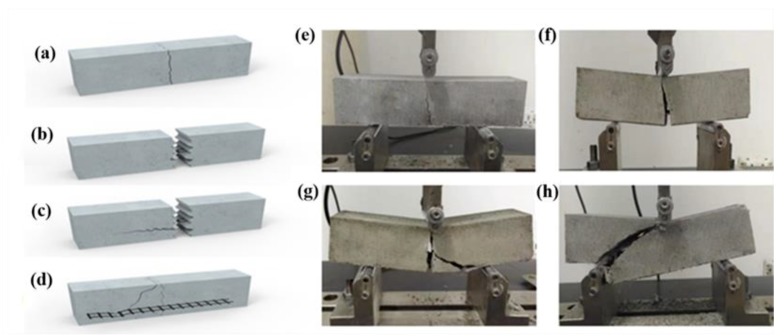
Different failure modes: (**a**,**e**) Mortar brittle fracture; (**b**,**f**) fiber slippage; (**c**,**g**) transverse crack propagation; (**d**,**h**) inclined crack and fiber rupture.

The load–displacement curves of composites are presented in Figure 8. When the composites were subjected to a three-point bending load, the cracks firstly appeared in the middle of the bottom surface of the specimen. The average value of anti-folding load of I-(F) was determined as 3.241 kN. The cracks expanded upwards as the load increased for I-(F). As shown in Figure 9a,e, the fracture of the specimen was flush with only a small energy loss. For I-CF(F) and I-CF/EP(F), the magnitude of the slope of the load–displacement curve indicated two rising phases: rapid growth and slow growth. The curve rose rapidly when the load of the sample did not reach the ultimate load. As shown in Table 4, the average values of the specimen’s flexural load were 3.526 and 3.910 kN, respectively. The curve immediately dropped following a steady growth when the load exceeded the ultimate load. Then, there was a slow rise for the curve, which was related to the resistance of the carbon fiber mesh inside the sample. As shown in Figure 9b,f, the slip of the carbon fiber mesh inside the sample could be the appropriate reason. For I-CF/EP-SCA(F), the anti-folding load average value of samples was 5.347 kN. The load–displacement curve of I-CF/EP-SCA(F) showed three different rising periods: rapid growth, slow growth, and slow growth. The first rapid growth occurred before the load of the sample reached the ultimate load. SCA increased the chemical connection between the carbon fiber mesh and the mortar, which caused the second rapid growth of the load–displacement curve. At this time, the carbon fiber mesh and mortar work together to suppress crack growth, so the load grows fast. However, the continuous expansion of the transverse crack caused the following decrease of the curve slope. Meanwhile, the crack expanded along the carbon fiber mesh while expanding upwards (Figure 9c,g). With the HMC added into the mortar matrix, the anti-folding load average value of II-HMC&CF/EP-SCA(F) increased to 8.097 kN. As shown in Table 4, the average flexural strength of I-(F) and II-HMC&CF/EP-SCA(F) were 7.60 and 18.82 MPa, respectively, whereby the flexural strength of II-HMC&CF/EP-SCA(F) increased by 147.6%, compared to I-(F). As shown in Figure 9d,h, inclined cracks and fiber rupture appeared in the sample due to increased bond strength at the interface between the carbon fiber mesh and mortar. The failure forms of the double shear tests and the three-point bending tests were not exactly the same, which is mainly caused by the difference in the load that the specimen is subjected to during the two tests. The carbon fiber mesh and mortar were mainly subjected to the tensile shear load and bending shear load in the two tests, respectively. For I-CF/EP(F), the carbon fiber mesh slipped due to the low bond strength between the epoxy coating and mortar when the specimens were subjected to bending shear load. For I-CF/EP-SCA(F), the crack was destroyed in the mortar layer along the direction in which the carbon fiber mesh is laid, with enhanced bonding ability between the carbon fiber mesh and mortar. The bond strength in the interface of the composite increased with the addition of HMC. Therefore, the main crack was an oblique crack and the carbon fiber broke when the specimens were subjected to bending shear load. Therefore, the three-point bending test was used to verify the effect of the interfacial shear strength of composites with SCA and HMC from another angle.

### 3.6. Load-Transfer Model Analysis

**Figure 10:**
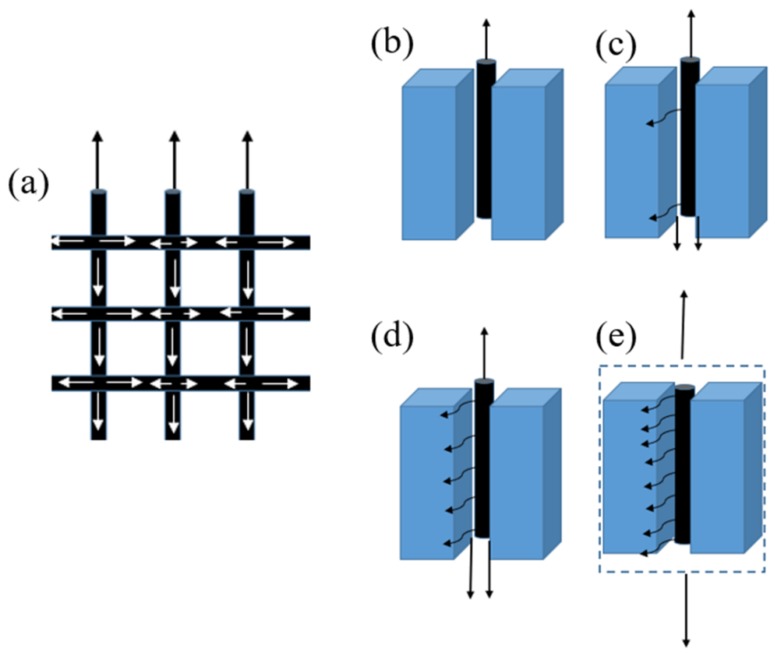
Load transfer diagram: (**a**) Carbon fiber mesh, (**b**) I-CF, (**c**) I-CF/EP, (**d**) I-CF/EP-SCA, and (**e**) II-HMC&CF/EP-SCA.

Figure 10 shows the load-transfer mode inside of the CMCCs when external force was applied. Because the carbon fiber mesh contains radial and latitudinal directions, whereby each direction is wound by two bundles of 12 k yarns, the load will be transmitted bidirectionally in the mesh direction when the external load acts on the carbon fiber mesh. For untreated carbon fiber meshes, it was extremely difficult to form an effective bond with the mortar at the interface due to the large surface inertness, so the external force acting on the carbon fiber mesh cannot be transferred to the interior of the mortar matrix (Figure 10a). With the deepening of the treatment of carbon fiber mesh with EP and SCA, the surface activity of the carbon fiber was enhanced (Figure 10b,c). At the same time, the load can be transferred to the mortar matrix more and more. The bonding effect between the mortar treated with HMC and the carbon fiber mesh after surface treatment was obviously improved. At this time, the system as a whole was subjected to external force, whereby the internal load was uniformly transmitted (Figure 10d).

## 4. Conclusions

The paper presents the results of double shear tests and three-point bending tests of CMCC composites based on different types of carbon fiber meshes, coupled with two types of mortar. It demonstrated the feasibility of fabricating CMCCs using SCA and HMC. The results revealed that after introducing SCA and HMC to the interface between carbon fiber mesh and mortar, the interfacial bonding of the composites could be significantly improved, which led to improved mechanical properties. For II-HMC&CF/EP-SCA, thanks to the increased number of C–OH bonds introduced by SCA and HMC, the carbon fiber mesh and mortar were integrated via a robust interface. The optimized interface resulted in a gradual transition between the uncracked and the cracked phase of the mortar and thus it considerably reduced the slip between the carbon fiber mesh and mortar. Thereby, the longitudinal shear strength of composites modified by SCA and HMC increased by 56.1%, compared to I-CF/EP. The II-HMC&CF/EP-SCA fabricated in this work is a promising composite material to be used for building reinforcement.

## Figures and Tables

**Table 1:** Detailed properties of materials.

Material/Mechanical Property	Value
**CF^1^**	
Ultimate breaking load	3200 N
Design tensile strength	101.2 MPa
Elastic modulus	230 kN/mm^2^
Areal density (force direction)	80 g/m^2^
Space	2 mm
**CWSM^1^**	
Compressive strength (28 Days)	55 MPa
Flexural strength	12 MPa
Splitting tensile bond strength	7 MPa
Density	2.05 g/cm^3^
**Clay Brick^2^**	
Compressive strength	19.8 N/mm^2^
Flexural strength	3.66 N/mm^2^
Splitting tensile strength	2.46 N/mm^2^
Young’s modulus	5760 N/mm^2^
**SCA^1^**	
Density	1.06 g/cm^3^
Refractive index	1.43 nD^25^
Content	98.4%
**HMC^1^**	
Viscosity	100,000

^1^ Specified by manufacturer,^2^ the data were obtained from [29].

**Table 2:** Experimental results of tensile properties tests.

Type of Composite	Specimen	F (KN)	$F_{av}$ (KN)	CoV(%)	$\sigma_{av}\left(\mathrm{MPa}\right)$	η
	S-1	0.98	1.09			
	S-2	1.07				
I-CF	S-3	1.23		8.3	690	0.19
	S-4	1.11				
	S-5	1.05				
	D-1	1.93				
	D-2	1.92				
I-CF	D-3	1.92	1.85	5.2	543	0.15
	D-4	1.79				
	D-5	1.71				
	T-1	3.44				
	T-2	3.31				
I-CF	T-3	3.26	3.40	3.4	665	0.18
	T-4	3.55				
	T-5	3.45				
	S-1	3.72				
	S-2	3.31				
I-CF/EP	S-3	3.54	3.53	4.2	2095	0.58
	S-4	3.55				
	S-5	3.53				
	D-1	6.31				
	D-2	6.27				
I-CF/EP	D-3	6.36	6.53	4.7	1943	0.54
	D-4	6.83				
	D-5	6.91				
	T-1	11.22				
	T-2	11.52				
I-CF/EP	T-3	11.73	11.51	2.2	2209	0.61
	T-4	11.31				
	T-5	11.78				
	S-1	4.42				
	S-2	4.39				
I-CF/EP-SCA	S-3	4.74	4.56	3.4	2667	0.74
	S-4	4.67				
	S-5	4.60				
	D-1	8.52				
	D-2	8.25				
I-CF/EP-SCA	D-3	8.67	8.50	1.8	2437	0.68
	D-4	8.52				
	D-5	8.52				
	T-1	14.80				
	T-2	14.13				
I-CF/EP-SCA	T-3	14.08	14.46	2.8	2809	0.78
	T-4	14.99				
	T-5	14.29				
	S-1	5.24				
	S-2	5.66				
II-HMC&	S-3	5.47	5.47	2.0	3185	0.88
CF/EP-SCA	S-4	5.37				
	S-5	5.44				
	D-1	10.08				
	D-2	10.17				
II-HMC&	D-3	10.55	10.33	2.0	2968	0.82
CF/EP-SCA	D-4	10.54				
	D-5	10.33				
	T-1	15.62				
	T-2	15.65				
II-HMC&	T-3	17.80	17.30	9.3	3602	1.00
CF/EP-SCA	T-4	18.25				
	T-5	19.21				

Note: The specimen is divided into two parts: x-xx, whereby x (S–single, D–double, T–triple) represents the number of carbon fiber meshes and xx represents the serial number of the sample.

**Table 3:** Surface element compositions of carbon fibers’ surface.

Samples	Atomic Composition (atm.%)		
	Carbon	Oxygen	Silicon
I-CF	85.63	14.37	-
I-CF/EP	83.09	16.91	-
I-CF/EP-SCA	81.23	17.03	1.74
II-HMC&CF/EP-SCA	68.62	31.05	0.33

**Table 4:** Experimental results of flexural performance tests.

Type of Composite	Specimen	F (KN)	$F_{av}$ (KN)	CoV(%)	$\sigma_{av}\left(\mathrm{MPa}\right)$	η
	1	3.28	3.24			
	2	3.34				
I-(F)	3	3.34		4.2	7.60	0.63
	4	3.21				
	5	3.01				
	1	3.53				
	2	3.63				
I-CF(F)	3	3.52	3.53	1.8	8.28	0.69
	4	3.47				
	5	3.48				
	1	3.90				
	2	3.91				
I-CF/EP(F)	3	3.98	3.91	1.0	9.18	0.7
	4	3.87				
	5	3.91				
	1	5.43				
I-CF/EP-SCA(F)	2	5.43				
	3	5.43	5.35	2.5	12.52	1.04
	4	5.34				
	5	5.12				
	1	8.12				
	2	7.93				
II-HMC&CF/EP-SCA(F)	3	8.06	8.09	2.4	18.82	1.57
	4	7.95				
	5	8.42				
